# Supplementary material for: Alginate Oligosaccharides Affect Mechanical Properties and Antifungal Activity of Alginate Buccal Films with Posaconazole
Source: Mar Drugs. 2019 Dec 9;17(12):692. doi: 10.3390/md17120692 (PMC6950700; doi:10.3390/md17120692)
Supplement: Supplementary file 1 [file marinedrugs-17-00692-s001.pdf]

marine drugs

Supplementary Materials

Alginate Oligosaccharides Affect Mechanical Properties and Antifungal Activity of Alginate Buccal Films with Posaconazole

Marta Szekalska [1,*], Magdalena Wróblewska [1], Monika Trofimiuk [1], Anna Basa [2] and Katarzyna Winnicka [1,*]

[1] Department of Pharmaceutical Technology, Medical University of Białystok, Mickiewicza 2C, 15-222 Białystok, Poland; magdalena.wroblewska@umb.edu.pl (M.W.); monikam@umb.edu.pl (M.T.)
[2] Institute of Chemistry, University of Białystok, Ciołkowskiego 1K, 15-245 Białystok, Poland
[*] Correspondence: marta.szekalska@umb.edu.pl (M.S.); kwin@umb.edu.pl (K.W.); Tel.: +48-85-748-5616 (M.S.)

Placebo Films Preparation by the Solvent-Casting Method

The films formulation S1-S9 were prepared by poured mixtures containing different concentrations of sodium alginate (ALG) and glycerol (used as plasticizer) into plexiglass moulds with the surface 14 × 14 cm and dried at 37 ± 1°C for 24 h. After drying, films were cut into pieces of 2 × 3 cm.

Placebo Films Preparation by the Freeze-Thaw Method

The films formulation F1-F9 were prepared according to the point *3.2.1* in the Article.

Table S1. Composition of designed alginate (ALG) films obtained by the solvent-casting (formulations S1-S9) and freeze-thaw (formulations F1-F9) method.

Formulation	ALG (g)	Glycerol (g)	Purified water (up to; g)	Thickness (μm)
**Films obtained by the solvent-casting method**				
**S1**	1	-	100	13.9±2.3
**S2**	1	0.3	100	16.5±3.5
**S3**	1	0.6	100	18.9±3.5
**S4**	2	-	100	29.8±3.3
**S5**	2	0.3	100	44.3±2.8
**S6**	2	0.6	100	63.3±7.1
**S7**	3	-	100	73.6±6.9
**S8**	3	0.3	100	80.7±6.9
**S9**	3	0.6	100	94.1±1.4
**Films obtained by the freeze-thaw method**				
**F1**	1	-	100	26.2±9.5
**F2**	1	0.3	100	26.5±6.5
**F3**	1	0.6	100	27.2±5.5
**F4**	2	-	100	37.3±5.5
**F5**	2	0.3	100	43.4±6.5
**F6**	2	0.6	100	58.8±6.7
**F7**	3	-	100	64.6±7.3
**F8**	3	0.3	100	70.6±4.2
**F9**	3	0.6	100	90.2±7.4

Mechanical properties

Mechanical properties (expressed by tensile strength) were examined according to the point *3.3.6.* in the Article.

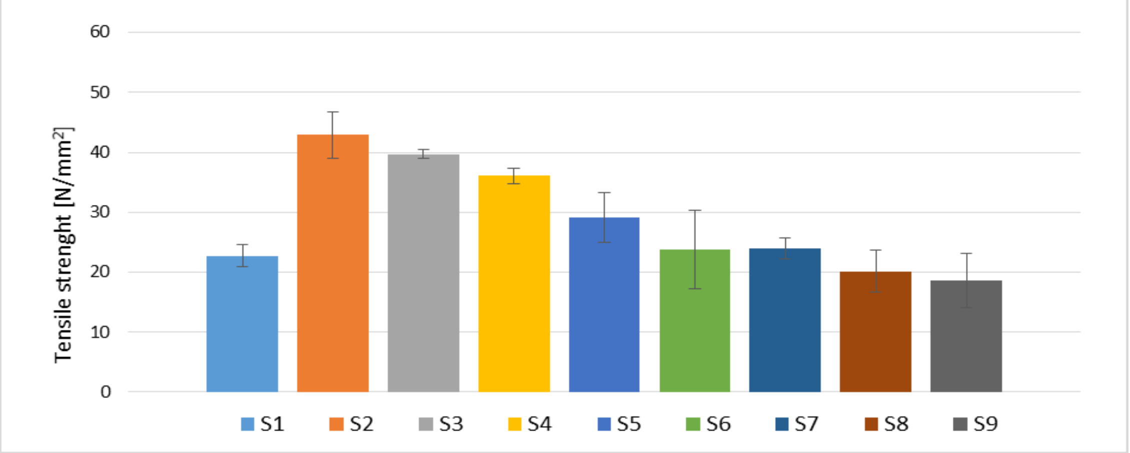

(a)

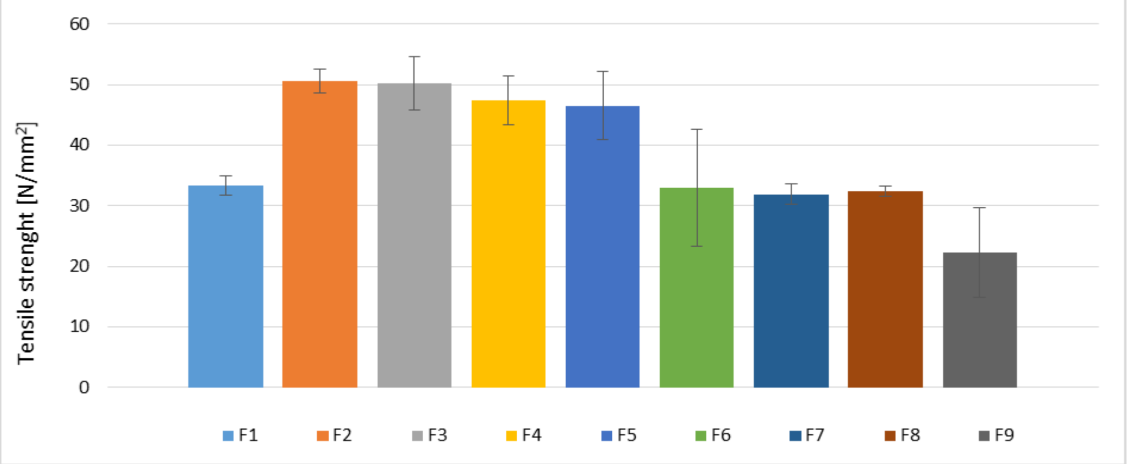

(b)

Figure S1. Tensile strength (TS) of films (a) obtained by the solvent-casting (formulations S1-S9) and (b) by the freeze-thaw (formulations F1–F9) method.

Swelling properties

Swelling properties were examined according to the description in the point *3.3.7.* in the Article.

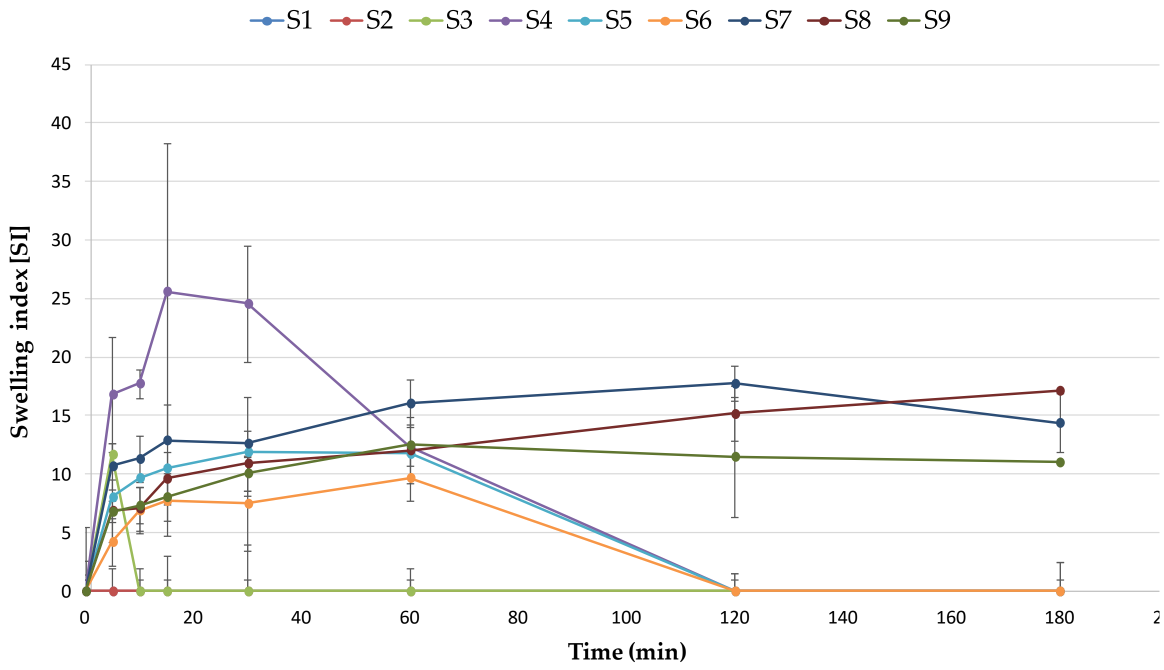

(a)

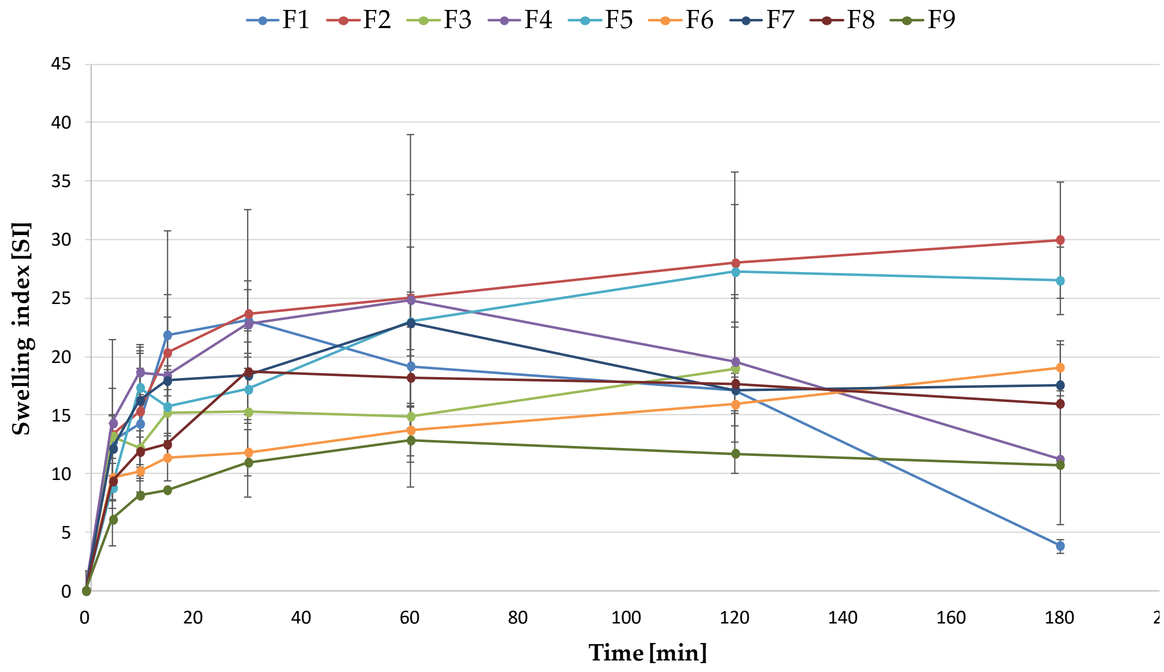

(b)

Figure S2. Swelling index (SI) of films (a) obtained by the solvent-casting (formulations S1-S9) and (b) by the freeze-thaw (formulations F1-F9) method.

Ex vivo mucoadhesive properties

Ex vivo mucoadhesive properties were examined according to the description in the point *3.3.8.1.* in the Article.

Table S2. Mucoadhesive properties of ALG films obtained by the solvent-casting method.

Formulation	F_{max}[N]	W_{ad}[μJ]
**S1**	0.46 ± 0.08	202.90 ± 18.43
**S2**	0.45 ± 0.10	177.23 ± 26.17
**S3**	0.43 ± 0.06	114.50 ± 10.01
**S4**	0.88 ± 0.09	240.43 ± 18.69
**S5**	0.83 ± 0.15	193.27 ± 45.32
**S6**	0.70 ± 0.14	150.63 ± 37.25
**S7**	1.42 ± 0.08	235.70 ± 21.56
**S8**	1.10 ± 0.15	211.47 ± 31.95
**S9**	0.92 ± 0.19	165.47 ± 24.52

Table S3. Mucoadhesive properties of ALG films obtained by the freeze-thaw method**.**

Formulation	F_{max}[N]	W_{ad}[μJ]
**F1**	0.86±0.07	295.82 ± 47.25
**F2**	0.55 ± 0.16	243.80 ± 10.98
**F3**	0.39 ± 0.18	175.33 ± 46.90
**F4**	1.33 ± 0.13	396.70 ± 10.49
**F5**	0.96 ± 0.06	287.56 ± 66.83
**F6**	0.41 ± 0.11	273.96 ± 11.46
**F7**	1.51 ± 0.20	457.20 ± 23.17
**F8**	1.45 ± 0.15	394.70 ± 31.38
**F9**	0.98 ± 0.18	350.67 ± 47.28
